# Endothelial inflammation and neutrophil transmigration are modulated by extracellular matrix composition in an inflammation-on-a-chip model

**DOI:** 10.1038/s41598-022-10849-x

**Published:** 2022-04-27

**Authors:** Rebecca B. Riddle, Karin Jennbacken, Kenny M. Hansson, Matthew T. Harper

**Affiliations:** 1 Department of Pharmacology, University of Cambridge, Cambridge, UK; 2 Bioscience Cardiovascular, Research and Early Development, Cardiovascular, Renal and Metabolism, R&D BioPharmaceuticals, AstraZeneca, Gothenburg, Sweden

**Keywords:** Lab-on-a-chip, Innate immunity, Inflammation, Acute inflammation

## Abstract

Inflammatory diseases are often characterised by excessive neutrophil infiltration from the blood stream to the site of inflammation, which damages healthy tissue and prevents resolution of inflammation. Development of anti-inflammatory drugs is hindered by lack of in vitro and in vivo models which accurately represent the disease microenvironment. In this study, we used the OrganoPlate to develop a humanized 3D in vitro inflammation-on-a-chip model to recapitulate neutrophil transmigration across the endothelium and subsequent migration through the extracellular matrix (ECM). Human umbilical vein endothelial cells formed confluent vessels against collagen I and geltrex mix, a mix of basement membrane extract and collagen I. TNF-α-stimulation of vessels upregulated inflammatory cytokine expression and promoted neutrophil transmigration. Intriguingly, major differences were found depending on the composition of the ECM. Neutrophils transmigrated in higher number and further in geltrex mix than collagen I, and did not require an *N*-formyl-methionyl-leucyl-phenylalanine (fMLP) gradient for transmigration. Inhibition of neutrophil proteases inhibited neutrophil transmigration on geltrex mix, but not collagen I. These findings highlight the important role of the ECM in determining cell phenotype and response to inhibitors. Future work could adapt the ECM composition for individual diseases, producing accurate models for drug development.

## Introduction

Inflammatory responses are required to successfully combat invasion by pathogens. However, excessive or unresolved inflammation can damage healthy tissue and result in chronic inflammatory conditions such as rheumatoid arthritis, ulcerative colitis, cardiomyopathies, cardiovascular disease, and asthma.

During an inflammatory response, neutrophils move from the blood to the site of inflammation by transmigrating across the endothelial barrier and through the basement membrane (BM) of the surrounding extracellular matrix (ECM). This multi-step process is enabled by inflammatory receptors and molecules expressed by the endothelial cells which capture the neutrophils and support their extravasation, alongside gradients of chemokines that guide the neutrophils through the ECM. The neutrophils respond by upregulating integrins that allow attachment to the endothelium and subsequently aid their movement through the BM^1^. These events must be tightly regulated to prevent excessive inflammation and tissue damage. Conversely, neutrophil transmigration is a potential drug target for treatment of chronic inflammatory conditions. However, crucial to developing novel therapies is a clear understanding of how neutrophil transmigration is regulated.

Neutrophil transmigration in vivo occurs in a 3D environment, and therefore typical 2D in vitro techniques cannot accurately recapitulate the process^2,3^. Static assays such as transwell migration in Boyden chambers^4^ are hindered by effects of gravity, lack of physiological flow, and difficulty in visualizing and analysing the neutrophils at each stage of transmigration. Equally, studying neutrophils using in vivo animal models has important ethical implications and can inaccurately model human physiology^5^. For example, in humans neutrophils comprise 50–70% of circulating leukocytes, compared to just 10–25% in mice^6^. Additionally, some cytokines found to play an important role in human inflammation such as IL-8^7^, are not expressed by mice. This lack of translation from current in vitro and in vivo models into humans has led to challenges in drug discovery^8^.

Organ-on-a-chip is a new and fast-growing field that has the potential to cross the translation gap and provide humanized models for use in both basic biology research and drug discovery. These computer chip-sized devices provide cells with a more physiological 3D environment that supports cell differentiation, enabling organ-like functions. The chips commonly consist of channels coated with ECM proteins and lined with one or more cell types. Additional physical factors such as shear flow, air–liquid interfaces, and cyclical deformation of the chips can also be incorporated^9,10^. With developments in stem cell biology, there is potential to derive cells from induced pluripotent stem cells of patient samples, increasing the feasibility of personalised medicine^11^. To reach this stage, models of different organs and biological processes and/or diseases need to be developed. Although the field is still relatively new, many organs have already been successfully modelled, including the lungs^12–14^, heart^15–17^, liver^18,19^, kidneys^20^, and skin^21–23^.

To model neutrophil transmigration during inflammation, the key components are the endothelial barrier and the ECM encountered post-transmigration. Neutrophils most commonly extravasate through post-capillary venules, which increase in permeability during inflammation. To recapitulate this, inflammatory mediators such as TNF-α are used to stimulate the endothelial barrier and induce production of inflammatory molecules that promote transmigration. After crossing the endothelium, neutrophils then encounter the basement membrane, a meshwork of proteins consisting primarily of collagen type IV, laminin, heparan sulfate proteoglycans, and nidogen^24,25^. Surrounding this, but further from the endothelial cells, is a fibrillar matrix containing collagen type I^26^. Neutrophil-ECM interactions occur directly through binding of neutrophil integrins to ECM proteins^27,28^ and indirectly via release of factors such as neutrophil proteases that remodel the ECM structure^29^. These interactions support neutrophil chemotaxis^30–32^ and prevent apoptosis^33,34^. Thus, the ECM is not simply a passive 3D scaffold but an active source of signals which sustains the inflammatory response^35^. Physiological ECM composition in organ-on-a-chip models is therefore important to understand how neutrophils navigate these matrices to reach the inflammatory site.

Although some organ-on-a-chip models have incorporated an endothelial component, this is usually a 2D sheet of endothelial cells on a porous membrane^12,21^ rather than a 3D vessel against a 3D ECM. A few groups have produced 3D neutrophil transmigration models^36–39^, investigating the role of chemoattractants, ECM pH, and endothelial cell type, but to our knowledge none have yet investigated the effect of ECM composition on endothelial and neutrophil responses to inflammation or inhibitors.

Here, we developed a novel model of neutrophil transmigration during inflammation, using the OrganoPlate system (Mimetas), a commercially available platform which has been used to model many organs already including the blood–brain barrier^40,41^, liver^42,43^, kidney^44,45^, and gut^46–48^, as well as the microvasculature^49–51^. The OrganoPlate consists of either 40 or 96 chips in a 384-well plate format, allowing the screening of many conditions in a more robust, reproducible, and high-throughput way than in-house manufacturing of individual devices. The OrganoPlate has been used to investigate the infiltration of neutrophils through a collagen I gel^52^ towards an inflamed intestinal tube, however this model did not include an endothelial component. It has also been used to study monocyte adhesion to the endothelium^51^, but no transmigration was observed in this model. Neither model considered the effect of the ECM on cell phenotype.

In this study, we used this platform to explore the effects of different ECM composition on trans endothelial neutrophil migration. This work demonstrates the importance of considering choice of ECM when designing organ-on-a-chip models. The multi-well plate format of the Organoplate enabled testing of anti-inflammatory compounds, supporting a use for the model in screening potential drug candidates for diseases involving excessive neutrophil infiltration and inflammation.

## Results

### HUVEC form confluent vessels in OrganoPlates on geltrex with 25% collagen I

**Figure 1:**
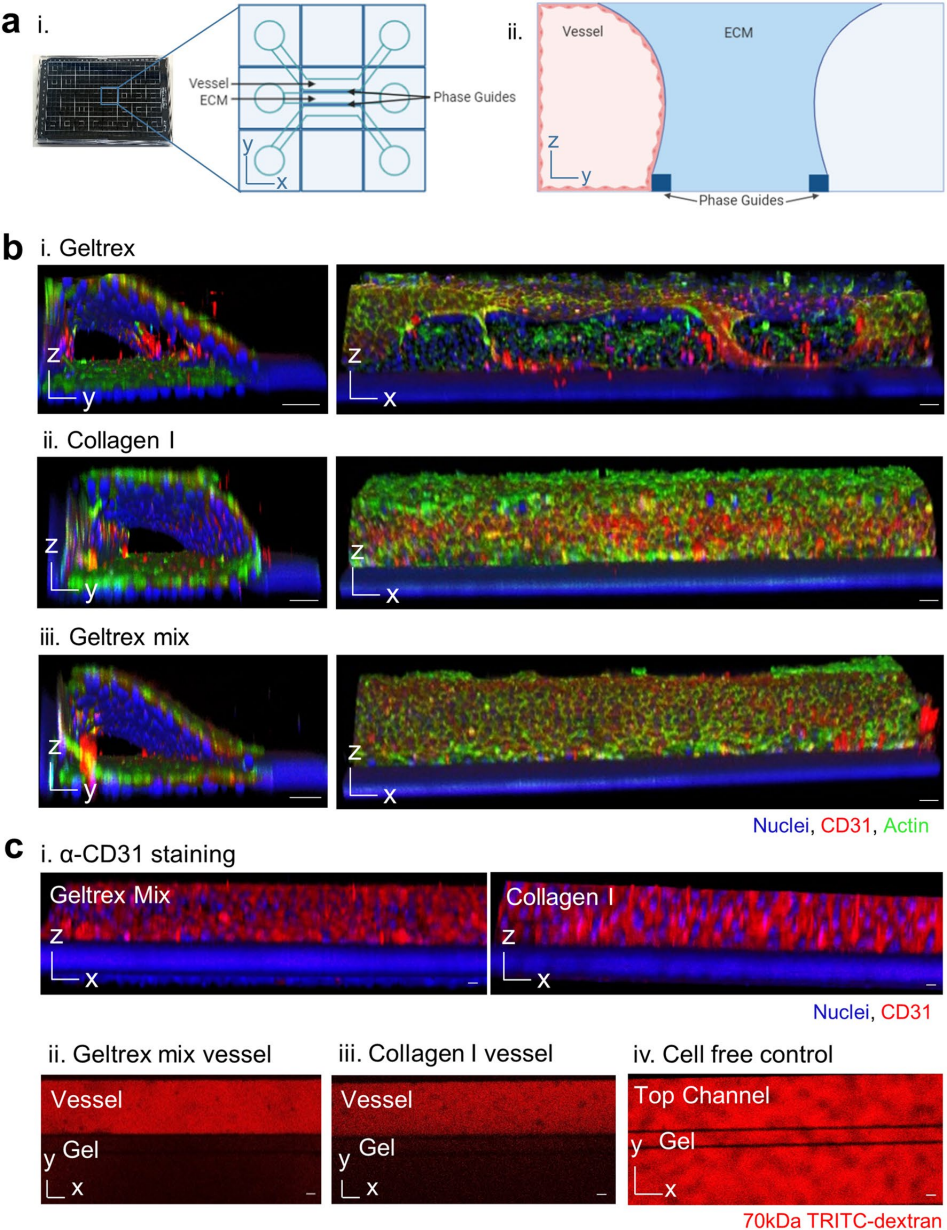
Incorporating collagen type I into geltrex extracellular matrix (ECM) enables formation of a leak-tight endothelial vessel in the 3-lane OrganoPlate. (**a**) 3-lane OrganoPlate schematics. i. The 3-lane OrganoPlate consists of 40 chips in a 384-well microplate format. Each chip has three lanes separated by two PhaseGuides™ which support patterning of hydrogels and cells via inlets and outlets. ii. Addition of hydrogel into the middle channel forms a barrier against which cells can be seeded in the top channel. Induction of flow via passive liquid levelling promotes vessel formation. The bottom channel is kept empty during vessel formation. (**b**) Vessel formation against different ECMs. ECM was introduced into the middle channel and incubated for 1 h to allow polymerisation. Human umbilical vein endothelial cells (HUVEC) were seeded into the top channel, left to adhere for two hours, and then placed on a plate rocker and incubated for 1 week. Vessels were fixed and then stained with Hoechst-33342 (blue), α-CD31 (red), and phalloidin-FITC (green). Images were taken on a Leica SP5 confocal microscope. 3D reconstructions of Z-stacks were produced in FIJI. Images are representative of at least N = 3 independent experiments. i. 14.6 mg/mL geltrex induced HUVEC tube formation against the ECM. ii. HUVEC formed confluent vessels against 4 mg/mL collagen I. iii. Incorporation of 0.75 mg/mL collagen I into 14.6 mg/mL geltrex (25:75) prevented tube formation and supported confluent vessel formation. (**c**) Vessels grown against geltrex + 25% collagen I exhibited physiological barrier function. i. HUVEC cultured in the 3-lane OrganoPlate formed intercellular junctions, as demonstrated by α-CD31 staining (red). ii. Mature vessels retained 70 kDa tetramethylrhodamine (TRITC)-dextran (red). 0.5 mg/mL TRITC-dextran was added to the vessel channel after 1 week of cell culture and incubated for 30 min. Dye retention was then imaged on a Leica SP5 confocal microscope. Scale bars are representative of 50 µm.

The neutrophil transmigration model was constructed in a Mimetas OrganoPlate 3-lane. The 3-lane OrganoPlate consists of a 384-well plate containing 40 chips, each with three channels. The channels are separated by Phaseguides, a liquid pinning technology which enables vessels to be grown against ECM, without any physical separation by membranes^53,54^ (Fig. 1a).

Two different ECMs, geltrex and collagen I, were compared in the OrganoPlate. Geltrex consists of a mix of proteins found physiologically in the endothelial basement membrane. However, when HUVEC were grown against geltrex alone, cells differentiated into tubes on the ECM, preventing proper vessel formation (Fig. 1b.i, Supplementary Movie 1). In contrast, when collagen I was used HUVEC grew on all four sides of the channel, forming confluent vessels (Fig. 1b.ii, Supplementary Movie 2), as previously reported^49,55–58^. To produce a more physiologically relevant basement membrane without stimulating differentiation, a mixture of 75% geltrex and 25% collagen I was tested (referred to as geltrex mix from here). This prevented the tube formation seen with geltrex alone (Fig. 1b.iii, Supplementary Movie 3). Successful vessel formation was confirmed by staining of intercellular junctions and ability of vessels to retain 70 kDa dextrans (Fig. 1c).

### TNF-α induces endothelial inflammation

**Figure 2:**
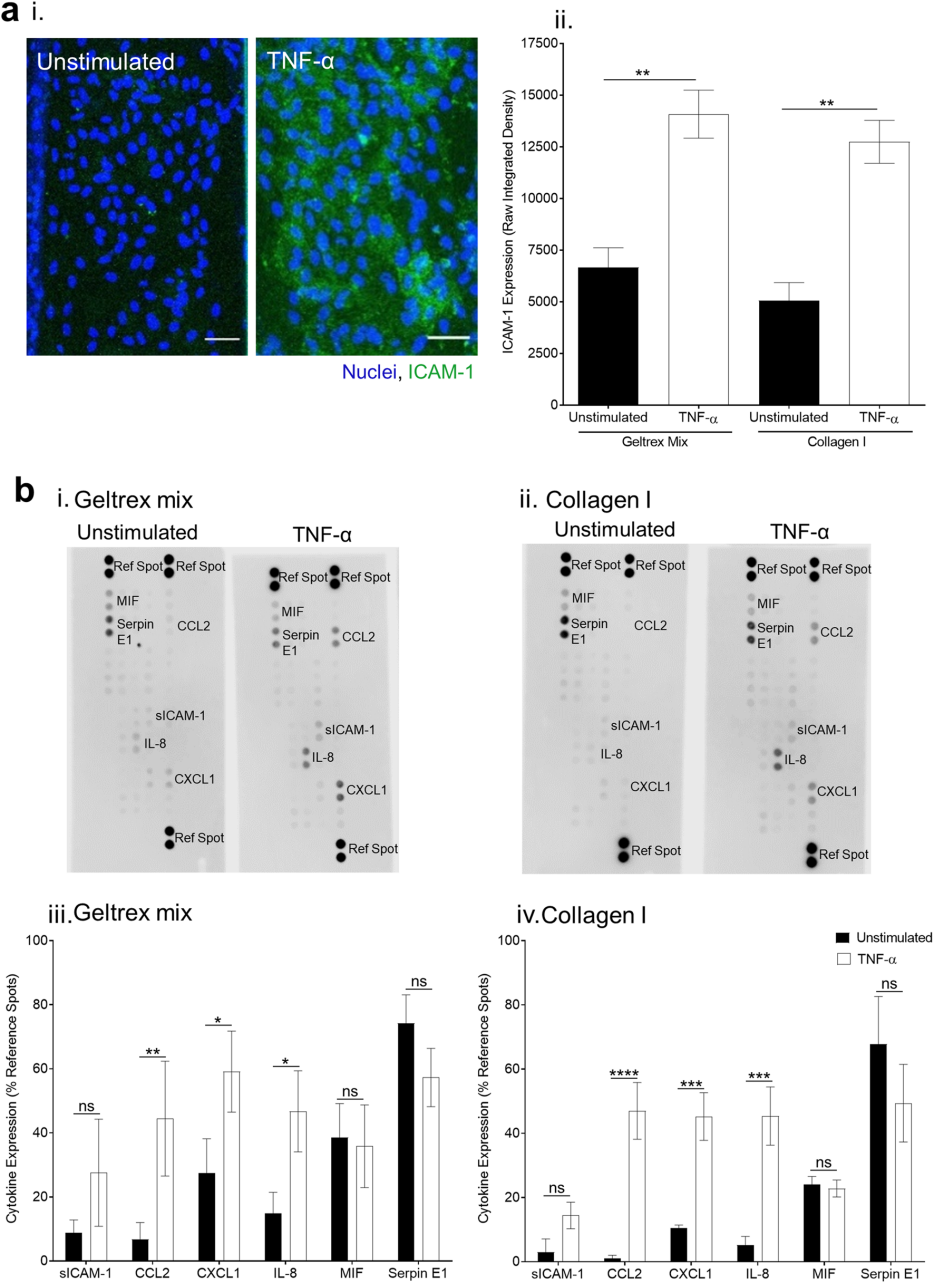
Tumour necrosis factor alpha (TNF-α) stimulation induces vessel expression of inflammatory molecules. (**a**) Stimulation of vessels with 1.6 ng/mL TNF-α induced Intercellular Adhesion Molecule 1 (ICAM-1) expression. i. Induction of ICAM-1 expression by TNF-α stimulation. Vessels were cultured for 1 week and then stimulated overnight with 1.6 ng/mL TNF-α. They were then fixed and stained with Hoechst-33342 (blue) and ICAM-1 (green). Images were obtained on a Leica SP5 confocal microscope. ii. Induction of ICAM-1 was quantified in FIJI. N = 5 independent experiments, mean ± SEM, **P < 0.01. (**b**) Inflammatory cytokines were released by vessels upon TNF-α stimulation. Supernatants were taken from vessels 24 h post-TNF-α treatment. Cytokine expression was analysed from 5 pooled vessels per condition using a Human Cytokine Proteome Profiler Array with LI-COR infrared detection. i. Representative images of unstimulated vs TNF-α stimulated cytokine arrays. ii. Densitometry was performed in FIJI. N = 3 independent experiments, mean ± SEM, ****P < 0.0001, ***P < 0.001, **P < 0.01, *P < 0.05, ns = non-significant. Scale bars are representative of 50 µm.

To develop an inflammation model, vessels were grown in the top channel with ECM patterned into the middle channel. Stimulation of confluent vessels with TNF-α upregulated expression of ICAM-1 on the vessel surface (Fig. 2a). Analysis of cytokine release into supernatants from stimulated vessels showed upregulation of several inflammatory cytokines: soluble intercellular adhesion molecule-1 (ICAM-1), interleukin-8 (IL-8), C–X–C Motif Chemokine Ligand 1 (CXCL1), and C–C Motif Chemokine Ligand 2 (CCL2) (Fig. 2b). In contrast, macrophage migration inhibitory factor (MIF) and serine protease inhibitor E1 (SERPIN E1) were present in both unstimulated and stimulated samples. These data show that an inflammatory state was successfully induced in the vessels. There was no significant difference in cytokine expression between collagen I and geltrex mix, in either unstimulated or TNF-α stimulated vessels (Supplementary Fig. S2).

### Neutrophils transmigrate upon inflammatory stimulation

**Figure 3:**
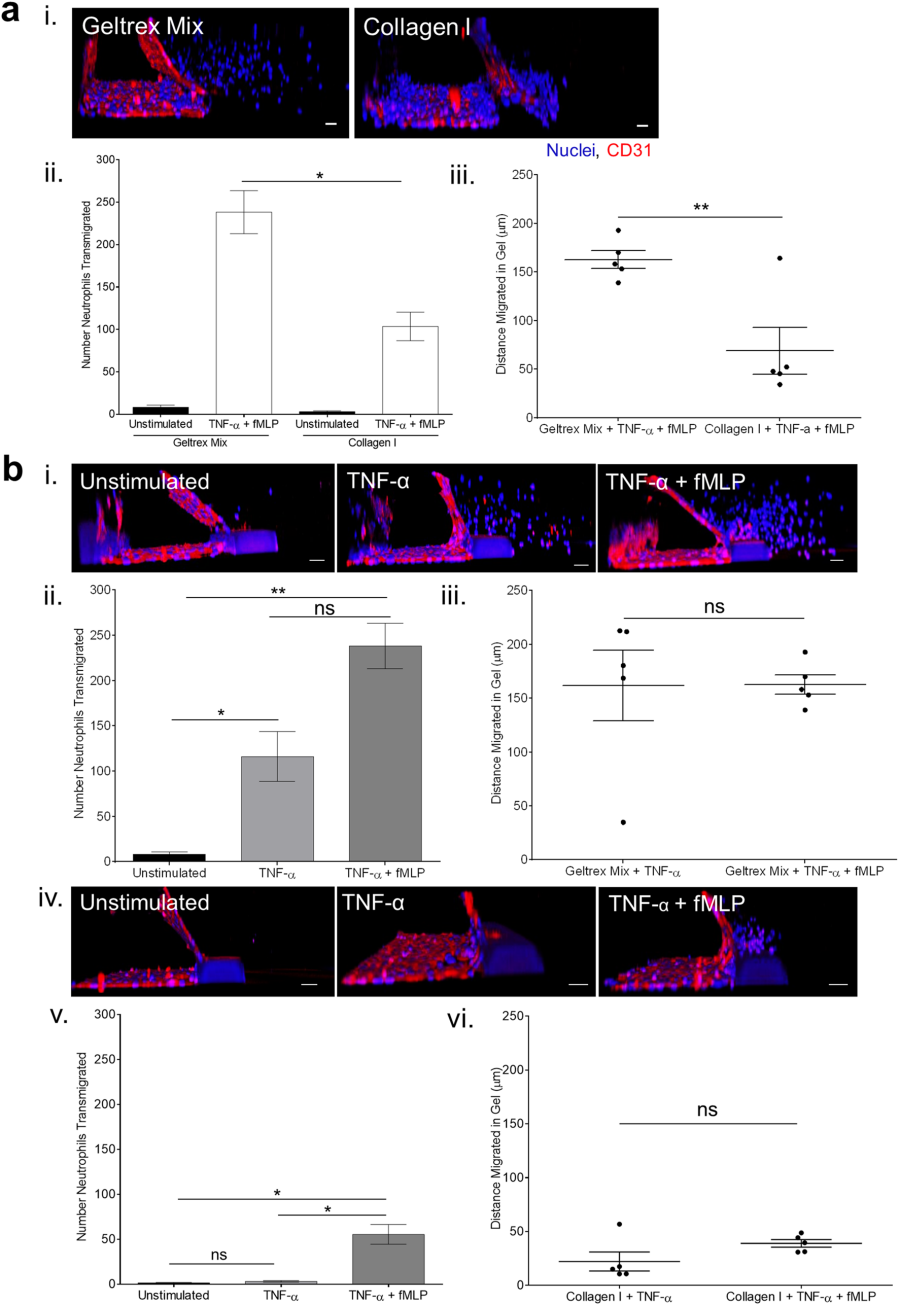
ECM composition affects characteristics of neutrophil transmigration across HUVEC vessels. (**a**) Transmigration of primary human neutrophils differs between geltrex mix and collagen I ECMs. Vessels were cultured for 1 week and then stimulated overnight with 1.6 ng/mL TNF-α. Neutrophils were isolated from human whole blood and added to the vessels for 1.5 h. *N*-formyl-methionyl-leucyl-phenylalanine (fMLP) was added to the bottom channel to generate a chemotactic gradient. Vessels were then fixed and stained with Hoechst-33342 (blue) and α-CD31 (red). Z-stacks were taken on a Leica SP5 confocal microscope. 3D reconstructions were generated in FIJI. Number/distance of neutrophils transmigrated was analysed using the Cell Counter plug in in FIJI. i. TNF-α stimulated HUVEC vessels supported neutrophil transmigration up an fMLP gradient. ii. More neutrophils transmigrated into geltrex mix than collagen I. iii. Neutrophils migrated further in geltrex mix than collagen I. (**b**) An fMLP chemotactic gradient was necessary for transmigration into collagen I but not geltrex mix. Neutrophil transmigration experiments were performed as described in part a, with or without the fMLP gradient present. i. Representative 3D reconstructions of neutrophil transmigration into geltrex mix. ii. In geltrex mix, presence of an fMLP gradient increased the number of neutrophils transmigrating. iii. Distance migrated in geltrex was not affected by fMLP. iv. Representative 3D reconstructions of neutrophil transmigration into collagen I. v. In collagen I, fMLP was required for neutrophils to transmigrate. vi. Distance migrated in collagen I was not affected by fMLP. N = 5 independent experiments/blood donors with n = 1–3 chips per condition, mean + /- SEM, ns = non-significant, *P < 0.05, **P < 0.01. Scale bars are representative of 50 µm.

Addition of freshly isolated human neutrophils into the inflamed vessels led to neutrophil transmigration out of the vessels and into the ECM (Fig. 3a.i). The use of human neutrophils increases the translational potential of this model, compared to use of immortalised cell lines. fMLP was added to the bottom channel to produce a gradient of chemoattractant for the neutrophils to migrate towards. Neutrophil transmigration was easily quantifiable by imaging due to the structure of the OrganoPlate, where the vessel and ECM are side by side. This allows straightforward analysis of both number of neutrophils transmigrated and the distance migrated within the ECM.

Very few neutrophils transmigrated across unstimulated vessels. Neutrophil transmigration differed between geltrex mix and collagen I ECMs. Fewer neutrophils transmigrated into collagen I, and post-transmigration neutrophils appeared unable to migrate towards the fMLP, stalling within 150 µm of the vessel. In contrast, neutrophils easily migrated throughout geltrex mix, covering the whole width of the gel channel during the 1.5-h experiment (Fig. 3a, Supplementary Fig. S3).

Interestingly, when the chemoattractant was not present, neutrophils still transmigrated into geltrex mix, although the number transmigrated was slightly reduced. In contrast, fMLP was required for transmigration into collagen I, with transmigration without fMLP comparable to unstimulated levels. The distance of migration in gel was not affected by presence of chemoattractant, in either geltrex mix or collagen I (Fig. 3b, Supplementary Fig. S3).

### Pharmacological inhibition of neutrophil transmigration depends on vessel ECM

To investigate the potential of our model for testing anti-inflammatory or immunomodulatory compounds, we investigated the effect of inhibiting neutrophil proteases. A cocktail of protease inhibitors was used; an elastase inhibitor (sivelestat), a cathepsin G inhibitor (Cathepsin G Inhibitor 1), and a dual elastase and cathepsin G inhibitor (human recombinant SLPI). These were first tested in HUVEC-free OrganoPlates, to isolate their effect on neutrophil migration into the ECM. In this experiment, the ECM was plated 1 day before neutrophil addition. fMLP was added directly before neutrophil perfusion.

**Figure 4:**
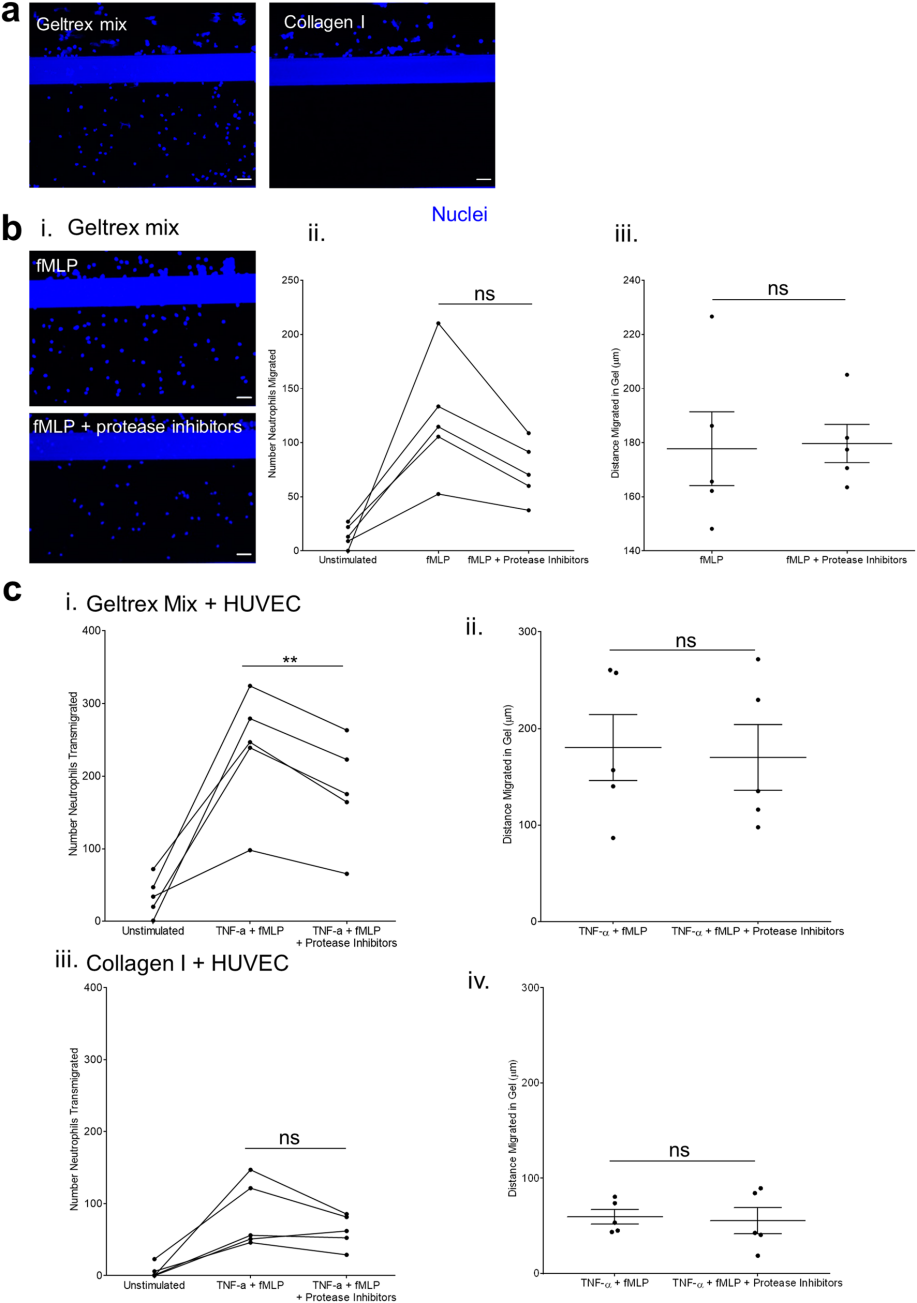
Inhibition of neutrophil proteases. (**a**) Neutrophils are unable to migrate into collagen I without presence of HUVEC vessel. Maximum projections of ECM Z stacks. ECMs were patterned into the middle channel and incubated overnight with media in the top channel. Neutrophils were isolated from whole blood and added to the vessels for 1.5 h. fMLP was added to the bottom channel to generate a chemotactic gradient. ECMs were fixed and nuclei stained with Hoechst-33342 (blue). Z-stacks were taken on a Leica SP5 confocal microscope. (**b**) Number of neutrophils migrating into geltrex mix is reduced by protease inhibitors but distance migrated in ECM is unaffected. ECMs were patterned into the middle channel and incubated overnight with media in the top channel. Neutrophils were isolated from whole blood and incubated with protease inhibitors for 15 min before being added to the vessels for 1.5 h. fMLP was added to the bottom channel to generate a chemotactic gradient. ECMs were fixed and nuclei stained with Hoechst-33342 (blue). Z-stacks were taken on a Leica SP5 confocal microscope. Number (ii)/distance (iii) of neutrophils migrated was analysed using the Cell Counter plug in in FIJI. c. Effect of protease inhibitors on neutrophil transmigration. Vessels were cultured for 1 week and then stimulated overnight with 1.6 ng/mL TNF-α. Neutrophils were isolated from whole blood and incubated with protease inhibitors for 15 min before being added to the vessels for 1.5 h. fMLP was added to the bottom channel to generate a chemotactic gradient. Vessels were then fixed and stained with Hoechst-33342 and α-CD31. Z-stacks were taken on a Leica SP5 confocal microscope. Number (ii,iii)/distance (iii,iv) of neutrophils migrated was analysed using the Cell Counter plug in in FIJI. N = 5 independent experiments/blood donors, n = 1–4 chips per condition, mean ± SEM, ns = non-significant, **P < 0.01. Scale bars are representative of 50 µm.

Neutrophils were unable to migrate into collagen I at all without HUVEC present (Fig. 4a). This suggests that the presence of HUVEC either makes the collagen I more permissible to migration, or that signalling between the HUVEC and neutrophils during transmigration activates the neutrophils and subsequently supports their migration. In contrast, neutrophils readily migrated into geltrex mix, when an fMLP gradient was present. This migration into geltrex mix was reduced by the protease inhibitor cocktail. The reduction seen was in numbers of neutrophils migrating rather than distance migrated in the ECM (Fig. 4b, Supplementary Fig. S4).

When HUVEC were incorporated into the model, number of neutrophils transmigrating into geltrex mix was inhibited by the protease inhibitor cocktail across (Fig. 4c). This inhibition was seen in all neutrophil donors tested. In contrast, this inhibition was not seen consistently in vessels grown against collagen I. Together, these data indicate that our inflammation-on-a-chip model can be used to explore the effect of potential immunomodulatory compounds Moreover, these results indicate that the effects of such immunomodulatory compounds can vary depending on the underlying ECM.

## Discussion

In this study we present a new 3D in vitro model of neutrophil transendothelial migration and ECM infiltration. Confluent endothelial vessels were successfully cultured against two different types of ECM. The endothelial cells were stimulated to mimic an inflammatory state, leading to primary human neutrophil transmigration into the ECM. Significant differences were found between neutrophil transmigration into the different ECMs, including differences in response to pharmacological inhibition. This work highlights the ECM as an important modulator of cell phenotype, and ECM composition should therefore be considered carefully when designing complex in vitro models.

The Mimetas 3-lane OrganoPlate was chosen as the platform for the model, as it allows culture of cells under perfusion and against a 3D ECM without any physical separation by membranes or barriers^53,54^. This is important when studying direct cell-ECM interactions and cell migration across the vessel wall. The three-lane structure allows the endothelium to be accessed both apically and basally. Therefore, inflammatory stimuli can be added in a physiologically relevant manner. For example, in our model we established an fMLP gradient across the ECM to mimic signals from the site of inflammation. A caveat to the use of this system is that the flow present is bidirectional rather than the physiological unidirectional. Previous studies have found that endothelial cells do not elongate or align in the direction of flow when it is bidirectional^59,60^, and express higher levels of inflammatory cytokines than when under unidirectional flow^60^. However, the presence of bidirectional flow is favorable over static as it allows the rolling of neutrophils over the endothelium, a key step in the process of transmigration in vivo^61^. In addition, the convenience of a pump free system and the 384-well plate format allows higher throughput experiments and enables testing of many conditions simultaneously, making this system attractive for drug screening.

ECM composition is a major regulator of neutrophil infiltration and yet physiologically relevant ECM are often overlooked in favour of experimentally convenient substrates. Two types of ECM were tested in the model, collagen I and geltrex. Collagen I is well established as a 2D coating or 3D ECM for endothelial cells, as it robustly supports adherence and monolayer formation^49,55–58^. In contrast, studies on culturing endothelial cells on basement membrane extracts (BMEs) such as geltrex or matrigel focus on induction of tube formation, modelling angiogenesis^62–64^. Our initial findings mirror these approaches, with successful vessel formation on collagen I, and vessel retraction and tube formation against geltrex. However, by combining geltrex with a small amount of collagen I we were able to create an ECM composition that supported vessel formation. Physiologically, the ECM consists of a thinner layer of basement membrane similar to geltrex, with a thicker layer of interstitial collagen I matrix below. Although our in vitro model does not recapitulate this this bilayer, we are able to successfully culture vessels on a mixed matrix, exposing endothelial cells to a wider range of ECM components.

The difference in endothelial phenotype between collagen I and BMEs is probably due to multiple factors, including differences in stiffness of the gels and protein composition. A previous study cultured HUVEC on polyacrylamide gels, and found that as stiffness of the gel decreased HUVEC tended to undergo tube formation, but maintained monolayer formation at a higher stiffness^65^. Other studies have found the basement membrane protein laminin to play an important role in determining monolayer vs tube formation^66–68^. The basement membrane therefore influences endothelial phenotype, and so 3D models on ECM components have an opportunity to better represent the physiological microenvironment than 2D cultures, or 3D models on a single type of matrix protein.

Inflammation was induced in the vessels using TNF-α, a commonly used inflammatory stimulus, although other inflammatory stimuli could be readily compared in future studies. Immunofluorescence staining of surface ICAM-1, a key receptor for neutrophil binding, showed a significant upregulation upon stimulation. Analysis of vessel supernatants found induction of four inflammatory cytokines—soluble ICAM-1, CXCL1, CCL2 and IL-8. These cytokines have established roles in neutrophil transmigration^69–72^ and inflammatory diseases^73–76^ in vivo. Therefore, TNF-α was an appropriate stimulus for our model.

When neutrophils were added to stimulated vessels, differences in transmigration were observed depending on the ECM used. The first major difference seen was that many more neutrophils transmigrated into geltrex mix than collagen I, suggesting that vessels are more permissive to transmigration on geltrex mix. There is very little data directly comparing transmigration across endothelium on substrates of different protein composition, likely due to the propensity of HUVEC to undergo tube formation when cultured on BMEs. Studies have found that increasing stiffness of the ECM increases neutrophil transmigration^77,78^, and that this corresponds to changes in stability of cell–cell junctions, alongside increased upregulation of inflammatory markers and vascular permeability^79–81^. However, we found no difference in surface ICAM-1 or cytokine induction between the ECMs. It is possible that the differences we observe between the ECMs are due to the different protein compositions of geltrex mix and collagen I. Geltrex mix contains many different ECM components and growth factors that could alter HUVEC phenotype independently of mechanotransduction, demonstrating the importance of ECM composition in modelling inflammation.

Following transmigration, ability of neutrophils to infiltrate the ECM was hindered in collagen I compared to geltrex mix. This could again be due to mechanical differences between the ECMs, such as stiffness or pore size. Several groups have compared the structure of polymerized collagen I to BME gels, finding that although BME gels have smaller pores they are more deformable than collagen I matrices^82,83^. Furthermore, it could be that geltrex mix contains more binding sites for the neutrophils and therefore supports their migration. Previous studies have found that binding of neutrophils to ECM proteins such as laminin and mindin promotes their chemotaxis towards the inflammatory site^84,85^.

We also explored the requirement for a chemoattractant gradient in the model. Absence of fMLP in chips with geltrex mix did not significantly affect numbers or distance of neutrophils transmigrated. In contrast, vessels grown against collagen I required the presence of fMLP for induction of neutrophil transmigration. Geltrex mix itself does not induce neutrophil infiltration, as no neutrophil migration was observed into geltrex mix without HUVEC or fMLP present. Therefore, the threshold for induction of transmigration differs between the ECMs, with transmigration into collagen I requiring an extra inflammatory signal to direct the neutrophils across the endothelium.

We next investigated a role for neutrophil proteases in navigating the ECM post-transmigration. Whereas some reports suggest that neutrophils move passively through the basement membrane, favouring ‘low expression regions’ with lower protein concentration^86–88^, other studies support a role for neutrophils in releasing proteases to actively degrade the BM^31,89–91^. We first observed that neutrophils were unable to penetrate a collagen I matrix without HUVEC present, which suggests a role for the endothelium in supporting migration into the ECM. This could be due to activation or priming of the neutrophils during transmigration. Previous studies have found that post-transmigration neutrophils have an altered phenotype, including increased motility^92^, increased CD11b/CD18 expression^92,93^, and an increased ability to migrate through pericyte layers^94^. Alternatively, it could be that the HUVEC alter the underlying ECM, either by remodelling or by depositing their own ECM during culture. The ability of endothelial cells to degrade the ECM during angiogenesis is well established^95–97^, but whether remodelling of the ECM occurs in mature vessels is more uncertain.

In contrast to collagen I, neutrophils easily migrated into geltrex mix without presence of a vessel. Moreover, inhibiting elastase and cathepsin G moderately reduced this infiltration, demonstrating a contribution from neutrophil proteases. When the inhibitors were tested with vessels present, a modest but consistent inhibition of migration was seen in geltrex mix vessels. This was not seen in vessels formed on collagen I. This suggests that elastase and cathepsin G are partially responsible for enabling migration in geltrex mix. Other proteases such as matrix metalloproteinases (MMPs) have also been found to play a role in neutrophil infiltration, and may also play a role here^98–100^. The lack of effect in collagen I could indicate that these proteases are not involved in collagen I degradation, although there is evidence that in cell-free systems that both enzymes are able to perform this function^101,102^.

Our results emphasise the role of the ECM in modulating cell phenotype and response to inhibitors. Previous studies, particularly in the field of cancer, have also found that 3D environment and ECM composition affect drug efficacy. For example, several groups have found cancer cells to be less responsive to chemotherapy in 3D cultures than in 2D monolayers^103,104^, with one finding that fibronectin as a substrate increased the lack of responsiveness compared to collagen I^103^, and another showing increased resistance to chemotherapy in biological matrices compared to synthetic matrices^104^. These studies highlight that the ECM is a source of both mechanical and biological signals. Therefore, typical 2D cell cultures cannot accurately be used to test responses to drugs. Complex 3D models which recapitulate the in vivo microenvironment are needed for drug discovery. For example, inflammatory sites are often characterised by increased matrix stiffness^105^, which has been found to increase inflammation^106^ and reduce drug efficacy^107^. Future models could tailor ECM composition to each organ, disease, or person. Creation of bespoke ECM compositions will enable development of accurate models to increase understanding of cell behaviour in disease, and support drug development.

## Conclusions

3D in vitro organ on a chip models have the potential to bridge the gap between current in vitro and in vivo models. Development of these models requires knowledge of the microenvironment being modelled, including the physical 3D structure and the cell types and signals present. Inflammatory diseases are often characterised by excessive neutrophil infiltration and associated ECM remodelling. Here, we have successfully established a neutrophil transmigration on a chip model where neutrophils transmigrate out of an endothelial vessel and through a 3D ECM environment, as they would in vivo. We found substantial differences in characteristics of neutrophil transmigration depending on the ECM used, including different responses to inhibitors. This work highlights how extracellular components influence cell behaviour, and demonstrates a need for accurate recapitulation of in vivo microenvironments when designing in vitro models. Further work is required to fine-tune ECM structures for use in models and to delineate exactly how the ECM alters cell phenotype.

## Materials and methods

All materials were obtained from Sigma (UK) unless specified otherwise.

All experiments involving human participants was reviewed by the Human Biology Research Ethics Committee, University of Cambridge, with fully-informed, written consent in accordance with the Declaration of Helsinki.

### HUVEC culture

Human umbilical vein endothelial cells (HUVEC, PromoCell, Germany) were cultured in Endothelial Growth Medium (Media, PromoCell) with 30 µg/mL gentamicin, in T25 or T75 tissue culture flasks. Cells were grown to confluence (37 °C, 5% CO_2_) and then passaged using PromoCell Detach Kit. HUVEC were used at passage 4.

### Extracellular matrix (ECM) compositions

For collagen I ECM, 5 mg/mL rat tail collagen type I (ibidi, Germany) was neutralised by mixing at a ratio of 8:1:1 with 1 M HEPES and 37 g/L NaHCO_3_ producing a 4 mg/mL solution. For geltrex mix ECM, Geltrex™ LDEV-Free Reduced Growth Factor Basement Membrane Matrix (Gibco, A1413302) was diluted to 14.6 mg/mL in media and then mixed at a ratio of 75:25 (v/v) with 3 mg/mL collagen I. 3 mg/mL collagen I was made by neutralisation of 5 mg/mL collagen I with 1 M NaOH and 7.5% (w/v) NaHCO_3_ as per the manufacturer’s protocol. For each batch of geltrex, preliminary screening experiments were performed to confirm successful vessel formation. Some batches of geltrex required additional coating of the ECM with 10 µg/mL fibronectin for 2 days to support HUVEC adhesion.

### OrganoPlate^®^ culture

Three-lane OrganoPlates^®^ (Mimetas, 4003-400B, Netherlands) were used. 50 µL Hanks’ Balanced Salt Solution (without calcium and magnesium, HBSS, Gibco) was added to the observation windows to prevent chips from drying out. 1.75 µL geltrex mix or 1.85 µL collagen I was added to the middle lane of the chip via the gel inlet (Fig. 1a). ECMs were polymerised for 10 min (37 °C, 5% CO_2_), then 50 µL HBSS was added to the gel inlet to prevent ECM dehydration. Chips were incubated for a further 50 min, after which HBSS was aspirated from the gel inlets.

HUVEC were seeded into the top channel by addition of 2 µL cell suspension at 2 × 10^7^/mL. Media was added to the top channel inlet and the plate incubated for 2 h on its side at a 70° angle (37 °C, 5% CO_2_) to allow cells to attach to the ECM. Media was then added to the top channel outlet and perfusion begun (8 min intervals, 7° angle) on the Mimetas OrganoFlow S plate rocker. Plates were incubated under perfusion for 7 days to enable optimal barrier function of the vessels. Media was refreshed every 2–3 days.

### Barrier Integrity Assay

After 7 days of incubation, the barrier function of the vessels was tested. 0.5 mg/mL 70 kDa tetramethylrhodamine (TRITC)-dextran was added to the top channel for 30 min. Dye retention was imaged on a Leica SP5 confocal microscope. Barrier function was quantified by measuring the fluorescence in each channel in FIJI^108^ and then calculating the ratio of fluorescence in the vessel channel versus fluorescence in the ECM channel.

### Neutrophil isolation

Blood was taken from volunteers by venepuncture into 3.8% (v/v) sodium citrate-containing vacutainers (Greiner Bio-One). The volunteers were healthy and free of medication for at least 10 days prior to venepuncture. Fully-informed, written consent was obtained in accordance with the Declaration of Helsinki, and use of blood was reviewed by the Human Biology Research Ethics Committee, University of Cambridge. Nine mL blood was layered onto 5 mL histopaque-1077 and then centrifuged (428*g*, 30 min, room temperature, no brake). All layers above the red blood cell pellet were aspirated and the pellet diluted 1:1 (v/v) with HBSS, and then further diluted 1:1 (v/v) with 2% (w/v) dextran. The resulting solution was left at room temperature for 45 min to allow red blood cells to sediment. The opaque top layer was removed and diluted 1:1 (v/v) with HBSS and then centrifuged (200*g*, 10 min, room temperature, medium braking/acceleration). The pellet was resuspended in 1 mL sterile, deionized water for 45 s to lyse residual red blood cells, then topped up to 15 mL with HBSS and centrifuged again (200*g*, 10 min, room temperature, medium braking/acceleration). The resulting neutrophil pellet was resuspended at 3.75 × 10^6^/mL in Roswell Park Memorial Institute (RPMI) 1640 medium containing 10% (v/v) foetal calf serum.

### Neutrophil transmigration in OrganoPlates^®^

On day 7 of culture, vessel lumens were stimulated with 1.6 ng/mL human recombinant tumour necrosis factor-alpha (TNF-α) (R&D Systems) overnight. Immediately before neutrophil perfusion, the bottom channels of the chips were filled with 200 nM *N*-formyl-methionyl-leucyl-phenylalanine (fMLP). Neutrophils were then added to the vessels for 1.5 h on the rocker (37 °C, 5% CO_2_). The number of independent neutrophil donors used are specified in the figure legends.

### Protease inhibitor treatments

A cocktail of 100 µM sivelestat (Biotechne), 10 µM Cathepsin G Inhibitor 1 (Cambridge Bioscience), and 250 nM human recombinant secretory leukocyte peptidase inhibitor (SLPI) (Biotechne) was used. Neutrophils were incubated with the inhibitors or 0.2% (v/v) DMSO vehicle control for 15 min at room temperature before addition to the OrganoPlate^®^.

### Immunofluorescence

Vessels were fixed with 4% (w/v) paraformaldehyde (Santa Cruz Biotechnology) for 15 min, washed twice for five minutes with PBS and then blocked with 2% (w/v) BSA for 30 min. Anti-CD31 antibody (Abcam, ab9498) in 2% (w/v) BSA was added and incubated overnight at room temperature on a plate rocker. Vessels were washed twice with PBS and then anti-mouse-647 (ThermoFisher) secondary antibody, Hoechst-33342 (ThermoFisher), and phalloidin-FITC (ThermoFisher) were added and incubated for 1.5 h at room temperature in the dark, on a rocker. Two more PBS washes were performed. Images were obtained on a Leica SP5 confocal microscope, using a 20× objective. 3D reconstructions were created in FIJI^109^. Neutrophil transmigration was quantified in FIJI using the Cell Counter plug in. A neutrophil was considered transmigrated if it was to the right of the endothelial barrier, which was identified by CD31 staining (Supplementary Fig. S1). Neutrophils co-localised with CD31 staining were not considered transmigrated.

### Cytokine arrays

R&D Systems Cytokine Proteome Profiler Arrays were used to investigate cytokine production. Supernatants were taken from five chips 24 h after TNF-α stimulation and pooled together. Cytokine arrays were carried out as per manufacturer’s instructions, with the modified protocol for detection using AlexaFluor™ 790 Streptavidin (ThermoFisher). A LI-COR Odyssey Fc was used for detection of arrays. Cytokine levels were quantified by densitometry in FIJI. Cytokine expression was expressed as a % of the positive reference spots for each array.

### Statistical analysis

Statistical analysis was performed in GraphPad Prism v9. All tests were carried out using matched analyses. Two-way ANOVA with Sidak’s multiple comparisons test was used to analyse differences in surface ICAM-1 levels, cytokine expression, or number of neutrophils transmigrated between collagen I and geltrex mix, and unstimulated versus TNF-α stimulated. One-way ANOVA with Tukey’s multiple comparisons test was used to compare differences in number of neutrophils transmigrated between unstimulated, TNF-α, and TNF-α + fMLP, and also to compare unstimulated, stimulated, and protease inhibitor-treated conditions. For differences in distance of neutrophil migration, paired two-tailed t tests were used. Values P < 0.05 were considered significant. In figure legends, N refers to independent experiments/neutrophil donors, and n refers to technical repeats (number of vessels) within donors.

## Supplementary Information

Supplementary Information 1.

Supplementary Video 1.

Supplementary Video 2.

Supplementary Video 3.

## Data Availability

Data are available from the corresponding author on reasonable request.
