# Supplementary material for: Endothelial inflammation and neutrophil transmigration are modulated by extracellular matrix composition in an inflammation-on-a-chip model
Source: Sci Rep. 2022 Apr 27;12:6855. doi: 10.1038/s41598-022-10849-x (PMC9046410; doi:10.1038/s41598-022-10849-x)
Supplement: Supplementary file 1 — Supplementary Information 1. [file 41598_2022_10849_MOESM1_ESM.docx]

**
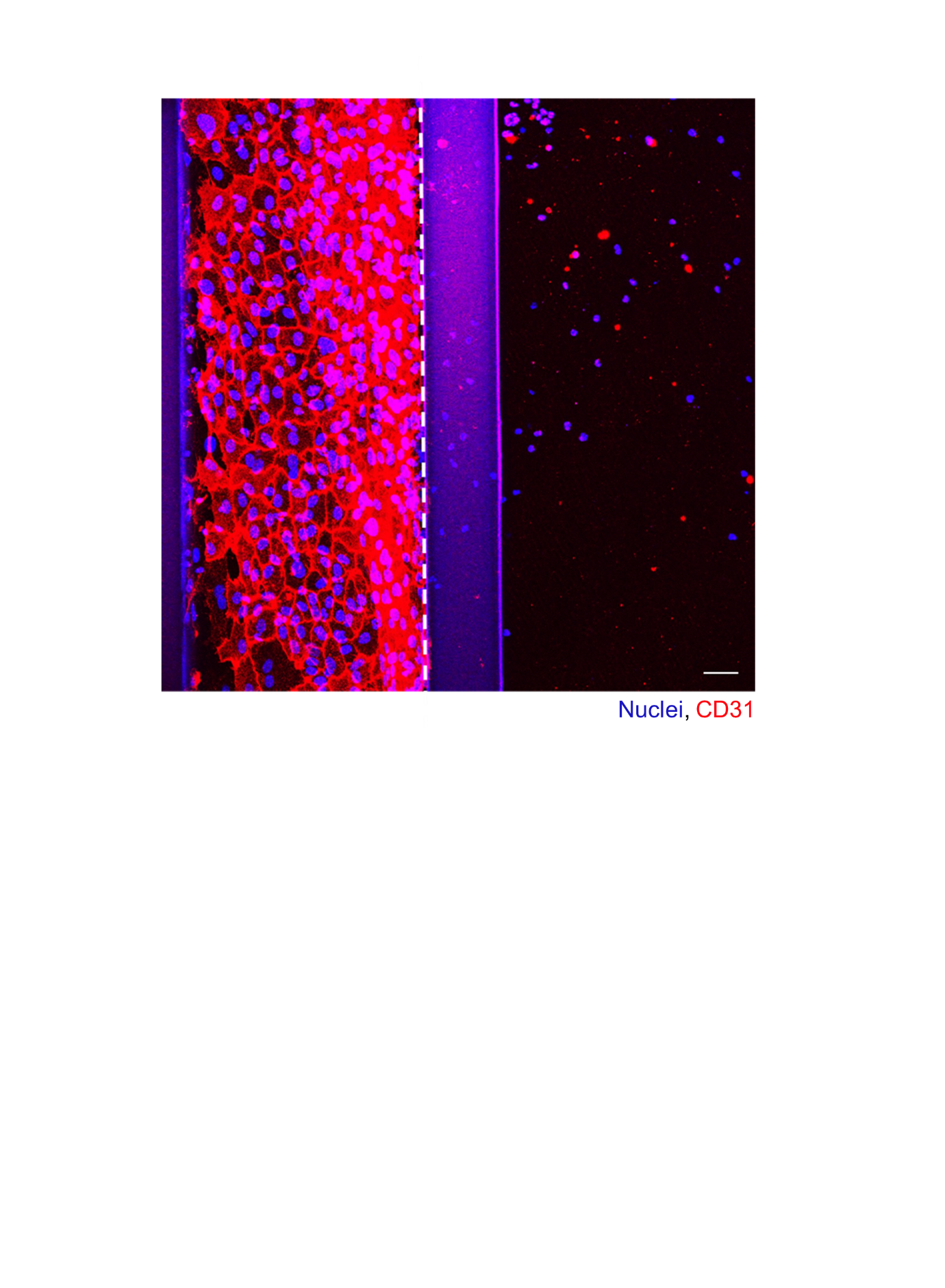
**

**Supplementary Figure S1**

**Quantification of Neutrophil Transmigration**

For image analysis of neutrophil transmigration, a neutrophil was considered transmigrated if it was to the right of the endothelial barrier (dashed white line). The endothelial barrier was identified by CD31 staining (red). The scale bar is representative of 50 µm.

**Supplementary Movies 1-3 of 3D Reconstructions of HUVEC Vessels Against Different ECMs**

ECM was introduced into the middle channel and incubated for 1 hour to allow polymerisation. Human umbilical vein endothelial cells (HUVEC) were seeded into the top channel, left to adhere for two hours, and then placed on a plate rocker and incubated for one week. Vessels were fixed and then stained with Hoechst-33342 (blue), α-CD31 (red), and phalloidin-FITC (green). Images were taken on a Leica SP5 confocal microscope. 3D reconstructions of Z-stacks were produced in FIJI. Images are representative of at least N=3 independent experiments.

**Supplementary Movie 1:** 14.6 mg/mL geltrex induced HUVEC tube formation against the ECM.

**Supplementary Movie 2:** HUVEC formed confluent vessels against 4 mg/mL collagen I.

**Supplementary Movie 3:** Incorporation of 0.75 mg/mL collagen I into 14.6 mg/mL geltrex (25:75) prevented tube formation and supported confluent vessel formation.

**
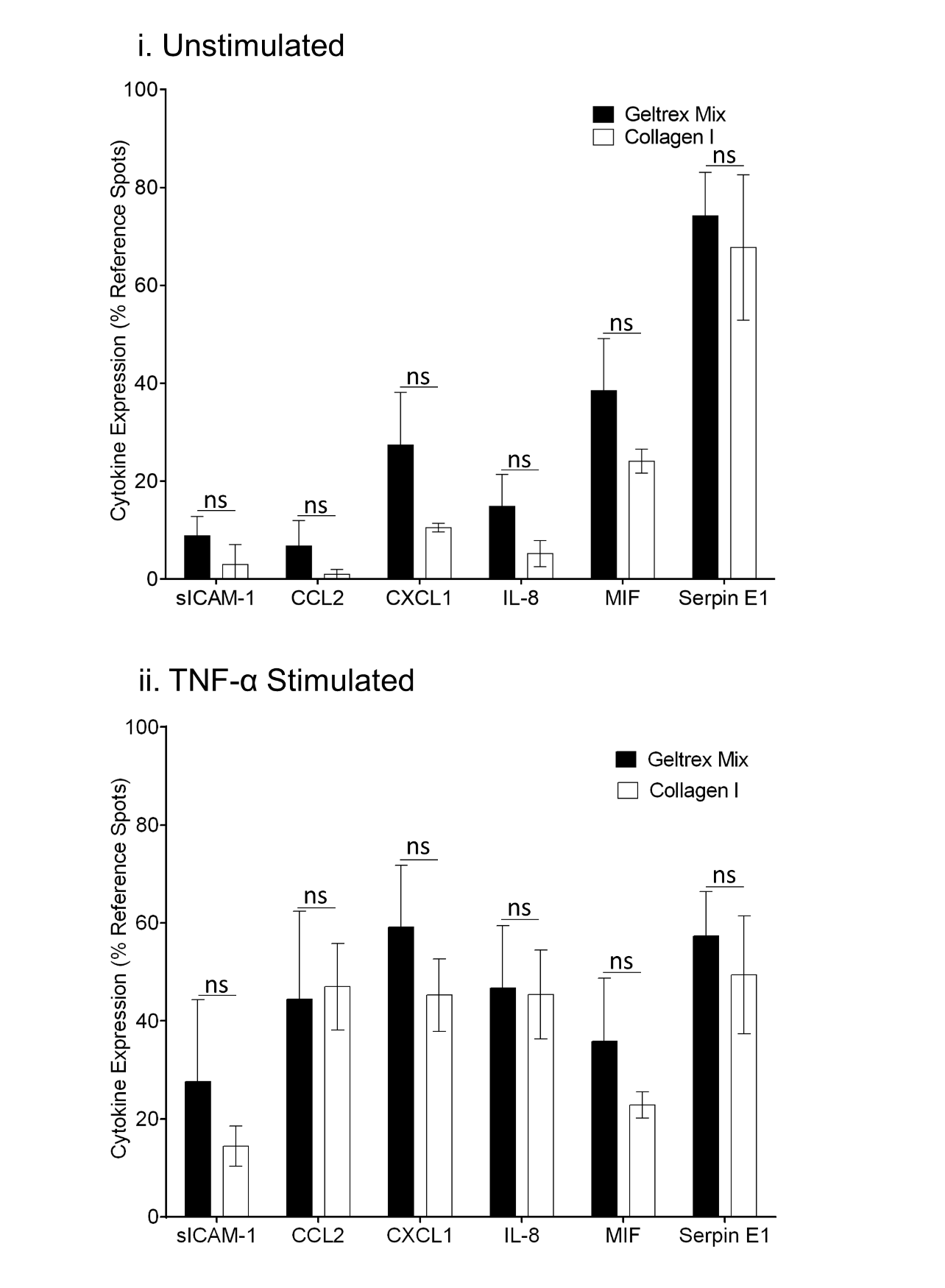
**

**Supplementary Figure S2**

**There are No Significant Differences in Cytokine Expression Between Collagen I and Geltrex Mix in Unstimulated or TNF-α Stimulated Vessels**

Two-way ANOVA analysis of cytokine expression between collagen I and geltrex mix in unstimulated (i) or TNF-α stimulated (ii) vessels. N=3 independent experiments, n=5 chips per condition, mean +/- SEM, ns = non-significant.

**
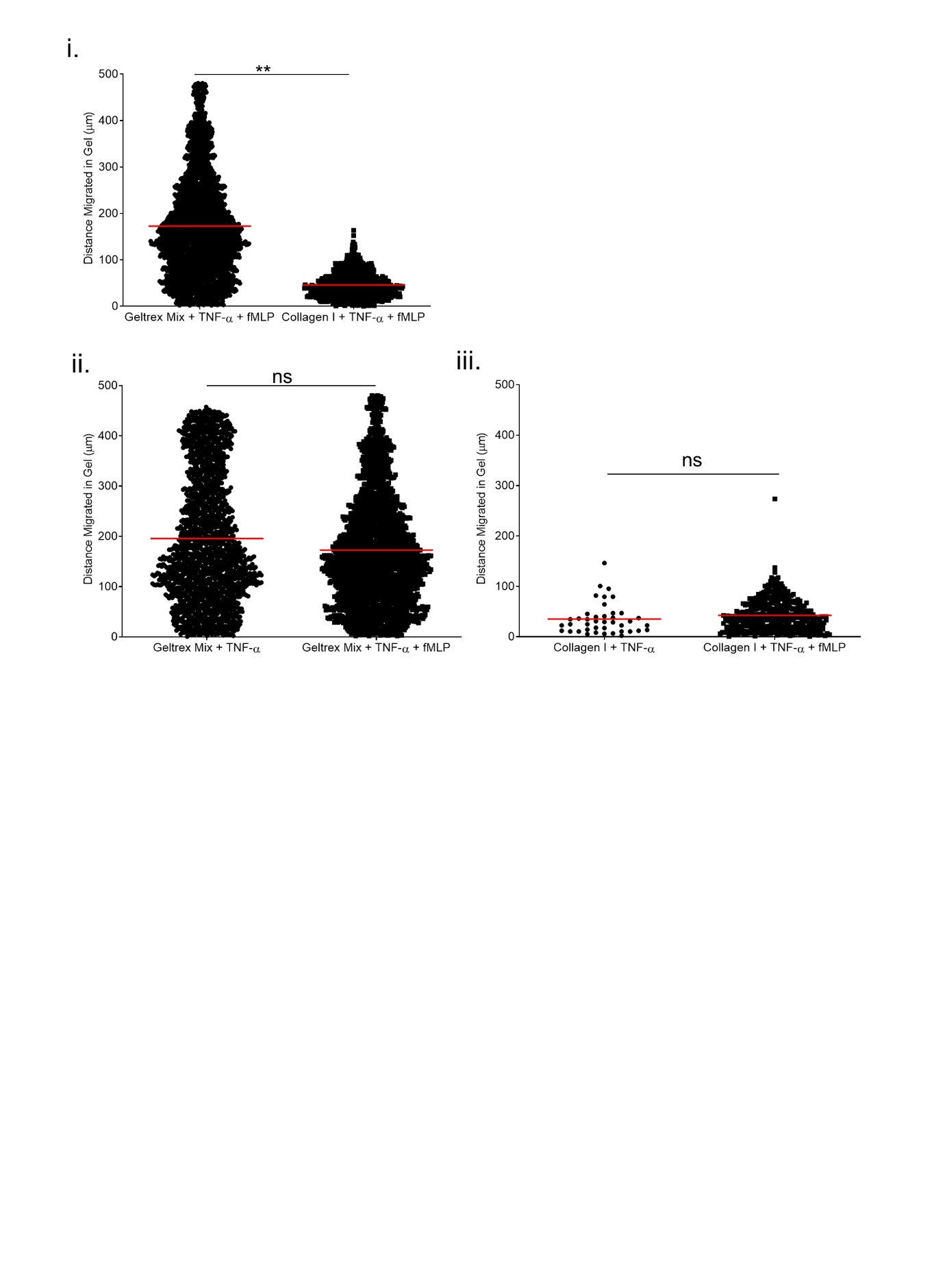
**

**Supplementary Figure S3**

**Distance migrated represented as individual neutrophils**

Vessels were cultured for one week and then stimulated overnight with 1.6 ng/mL TNF-α. Neutrophils were isolated from human whole blood and added to the vessels for 1.5 hours. *N*-formyl-methionyl-leucyl-phenylalanine (fMLP) was added to the bottom channel to generate a chemotactic gradient. Vessels were then fixed and stained with Hoechst-33342 and α-CD31. Z-stacks were taken on a Leica SP5 confocal microscope. Distance of neutrophils transmigrated was analysed using the Cell Counter plug in in FIJI. N=5 independent experiments/blood donors with n=1-3 chips per condition, mean +/- SEM, ns = non-significant, **P<0.01.


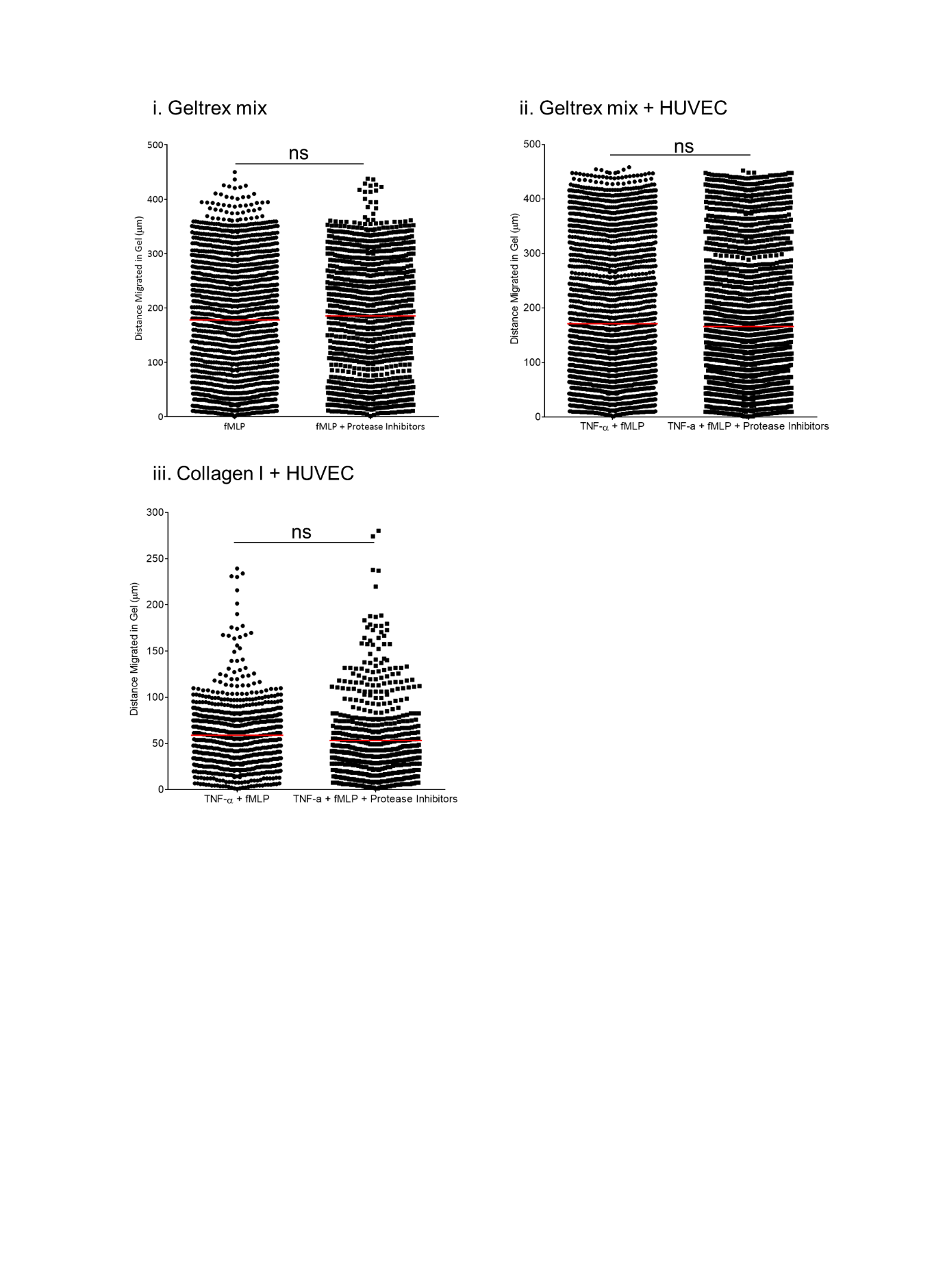


**Supplementary Figure S4**

**Distance migrated represented as individual neutrophils for protease inhibitor experiments**

Vessels were cultured for one week and then stimulated overnight with 1.6 ng/mL TNF-α. Neutrophils were isolated from whole blood and incubated with protease inhibitors for 15 minutes before being added to the vessels for 1.5 hours. fMLP was added to the bottom channel to generate a chemotactic gradient. Vessels were then fixed and stained with Hoechst-33342 and α-CD31. Z-stacks were taken on a Leica SP5 confocal microscope. Distance of neutrophils migrated was analysed using the Cell Counter plug in in FIJI. N=5 independent experiments/blood donors, n=1-4 chips per condition, mean +/- SEM, ns = non-significant.
